# Prevalence and mechanism of triazole resistance in *Aspergillus fumigatus* in a referral chest hospital in Delhi, India and an update of the situation in Asia

**DOI:** 10.3389/fmicb.2015.00428

**Published:** 2015-05-08

**Authors:** Anuradha Chowdhary, Cheshta Sharma, Shallu Kathuria, Ferry Hagen, Jacques F. Meis

**Affiliations:** ^1^Department of Medical Mycology, Vallabhbhai Patel Chest Institute, University of DelhiDelhi, India; ^2^Department of Medical Microbiology and Infectious Diseases, Canisius-Wilhelmina HospitalNijmegen, Netherlands; ^3^Department of Medical Microbiology, Radboud University Medical CenterNijmegen, Netherlands

**Keywords:** triazole resistant *A. fumigatus*, TR_34_/L98H, G54E, microsatellite typing, India, Asia

## Abstract

*Aspergillus fumigatus* causes varied clinical syndromes ranging from colonization to deep infections. The mainstay of therapy of *Aspergillus* diseases is triazoles but several studies globally highlighted variable prevalence of triazole resistance, which hampers the management of aspergillosis. We studied the prevalence of resistance in clinical *A. fumigatus* isolates during 4 years in a referral Chest Hospital in Delhi, India and reviewed the scenario in Asia and the Middle East. *Aspergillus* species (*n* = 2117) were screened with selective plates for azole resistance. The isolates included 45.4% *A. flavus*, followed by 32.4% *A. fumigatus*, 15.6% *Aspergillus* species and 6.6% *A. terreus.* Azole resistance was found in only 12 (1.7%) *A. fumigatus* isolates. These triazole resistant *A. fumigatus* (TRAF) isolates were subjected to (a) *calmodulin* and β *tubulin* gene sequencing (b) *in vitro* antifungal susceptibility testing against triazoles using CLSI M38-A2 (c) sequencing of *cyp51A* gene and real-time PCR assay for detection of mutations and (d) microsatellite typing of the resistant isolates. TRAF harbored TR_34_/L98H mutation in 10 (83.3%) isolates with a pan-azole resistant phenotype. Among the remaining two TRAF isolates, one had G54E and the other had three non-synonymous point mutations. The majority of patients were diagnosed as invasive aspergillosis followed by allergic bronchopulmonary aspergillosis and chronic pulmonary aspergillosis. The Indian TR_34_/L98H isolates had a unique genotype and were distinct from the Chinese, Middle East, and European TR_34_/L98H strains. This resistance mechanism has been linked to the use of fungicide azoles in agricultural practices in Europe as it has been mainly reported from azole naïve patients. Reports published from Asia demonstrate the same environmental resistance mechanism in *A. fumigatus* isolates from two highly populated countries in Asia, i.e., China and India and also from the neighboring Middle East.

## Introduction

Among the *Aspergillus* species, *Aspergillus fumigatus* is the leading etiologic agent of all forms of aspergillosis, which could be attributed to the ubiquitous presence of its thermo-tolerant spores that are refractory to adverse environmental conditions (Kwon-Chung and Sugui, 2013). *A. fumigatus*, in contrast to *Candida albicans*, has no reservoir in the immunocompetent population; thus, infections with *A. fumigatus* are generally environmentally acquired (Verweij et al., 2009; Chowdhary et al., 2013b). Furthermore, aspergillosis is associated with high morbidity and mortality in both immunocompetent and immunosuppressed populations primarily due to difficulties in early diagnosis or delay in recovery of the immune system (Kosmidis and Denning, 2015). Triazole antifungals, the competitive inhibitors of *cyp51A*, are preferred for prophylaxis and treatment of aspergillosis. However, failure of treatment with azoles and a steady increase in the occurrence of triazole resistant *A. fumigatus* (TRAF) isolates from environment as well as clinical settings has been reported (Denning et al., 1997; Mellado et al., 2007; Verweij et al., 2007; Rodriguez-Tudela et al., 2008; Snelders et al., 2008, 2009; Baddley et al., 2009; Howard et al., 2009; Arendrup et al., 2010; Mortensen et al., 2010, 2011; van der Linden et al., 2011, 2013; Burgel et al., 2012; Chowdhary et al., 2012a,b, 2013b, 2014a,b; Alastruey-Izquierdo et al., 2013; Badali et al., 2013; Bader et al., 2013; Escribano et al., 2013; Seyedmousavi et al., 2013; Prigitano et al., 2014; Spiess et al., 2014; Lavergne et al., 2015; Steinmann et al., 2015). The most common mechanism of triazole resistance has been linked to theTR_34_/L98H mutation with tandem repeat in the *cyp*51A promoter region combined with a single amino acid exchange of leucine 98 to histidine (Chowdhary et al., 2014c). Apparently, this mutated allele has spread throughout the *A. fumigatus* population and thus has been reported worldwide from patients as well as the environment (Mellado et al., 2007; Verweij et al., 2007; Rodriguez-Tudela et al., 2008; Snelders et al., 2008, 2009; Baddley et al., 2009; Howard et al., 2009; Mortensen et al., 2010, 2011; Lockhart et al., 2011; van der Linden et al., 2011; Burgel et al., 2012; Chowdhary et al., 2012a,b; Hamprecht et al., 2012; Jeurissen et al., 2012; Morio et al., 2012; Rath et al., 2012; Alastruey-Izquierdo et al., 2013; Bader et al., 2013; Escribano et al., 2013; Rocchi et al., 2014; Kidd et al., 2015; Steinmann et al., 2015). In addition several point mutations such as G54, G138, or M220 lead to disturbances in the docking of azole drugs to *cyp*51A protein rendering azole resistant *A. fumigatus* phenotype (Diaz-Guerra et al., 2003; Mann et al., 2003; Mellado et al., 2004; Chen et al., 2005; Howard et al., 2006, 2009, 2013; Rodriguez-Tudela et al., 2008; Snelders et al., 2008; Albarrag et al., 2011; van der Linden et al., 2011). These mutations have been previously reported to occur *de novo* due to prolonged exposure of *A. fumigatus* isolates to azole antifungal drugs in clinical settings (Chen et al., 2005; Howard et al., 2009; Escribano et al., 2012). However, a recent study reports presence of *A. fumigatus* carrying G54 point mutation in the environment of Tanzania, Romania, and India suggesting that environment may predominately be the cause in acquisition of azole resistant isolates (Sharma et al., 2015). Also, non-*cyp51A* mediated mutations have been increasingly recognized in the development of azole resistance and are mainly reported from Manchester, UK (Bueid et al., 2010). We conducted a prospective study for the assessment of prevalence of TRAF and the underlying *cyp51A* mutations in clinical isolates of *Aspergillus* species collected during a 4-year (2011–2014) period in a referral Chest Hospital in Delhi, India and reviewed the reports on TRAF isolates from environmental and clinical sources from Asia and the neighboring Middle East.

## Materials and Methods

### Fungal Isolates and Their Phenotypic Characterization

During 2011–2014 a total of 8222 clinical samples were processed for fungal culture and microscopy collected from patients of our hospital and three neighboring referral hospitals included in **Table 1**. The clinical specimens included sputum, endotracheal aspirates, bronchial aspirates, bronchoalveolar lavages (BAL), bronchial tissues, fine needle aspiration biopsies, lung biopsies, nasal polyps, bone marrow aspirations, pleural fluid, bronchial plugs, and cerebrospinal fluid. The study was approved by the Institute’s Ethics Committee. All *Aspergillus* species cultured from the specimens were preliminarily identified based on colony color and morphology of the isolates on Czapek dox agar plates incubated at 28∘C for 7 days. In order to investigate azole resistance in all of the *Aspergillus* species, they were screened on itraconazole (ITC, 4 μg/ml) and voriconazole (VRC, 1 μg/ml) supplemented Sabouraud dextrose agar (SDA) plates. *Aspergillus* isolates that exhibited growth on either of the antifungal plate were confirmed as *Aspergillus* species by amplification and sequencing of β*-tubulin* and *calmodulin* genes.

**Table 1:** Clinical characteristics of 12 patients with triazole resistant *Aspergillus fumigatus* (TRAF) isolates.

Patient no.	Age/sex/year of isolation	Specimen	Institution	MICs (μg/ml)^a,b^				Mutation	*Aspergillus* disease	Underlying condition	Treatment	Outcome
				ITC	VRC	ISA	POS					
1	60/M/2011	FNAB^c^/sputum	VPCI^d^	>16	≥8	≥8	≥8	TR_34_/L98H	IPA^e^	COPD^f^, Diabetes mellitus	VRC	Alive
2	65/M/2012	Lung biopsy	VPCI	>16	≥8	2	≥8	TR_34_/L98H	IPA	Pulmonary adenocarcinoma	VRC	Died
3	38/F/2012	FNAB	Hospital 1^g^	>16	≥8	2	≥8	TR_34_/L98H	IPA	Myelodysplastic syndrome	VRC	Died
4	19/F/2012	Endotracheal aspirate/tissue aspirate	VPCI	>16	8	8	1	TR_34_/L98H	IPA	COPD, Tuberculosis	AMB	Died
5	26/F/2012	Resected sinus tissue	Hospital 2^h^	≥16	>16	8	2	TR_34_/L98H	IA rhino- cerebral sinusitis	COPD	AMB	Died
6	52/M/2013	BAL^i^	Hospital 3^j^	>16	0.25	0.25	1	G54E	CPA^k^	Preexisting tubercular cavities	VRC	Died
7	76/M/2014	Sputum	VPCI	16	16	>8	4	TR_34_/L98H	ABPA^l^		Systemic steroids	Died
8	55/M/2014	Bronchial aspirate	VPCI	16	≥8	≥8	1	TR_34_/L98H	ABPA	-	Systemic steroids	No follow up
9	50/M/2014	BAL	VPCI	16	≥8	≥8	1	TR_34_/L98H	CPA	Tuberculosis	VRC	Died
10	50/M/2014	BAL	VPCI	16	2	4	0.06	F46Y, D255E, M172V	CPA	-	AMB for 1 month, discharged on VRC	No follow up
11	57/M/2014	Sputum	VPCI	≥16	>16	>8	2	TR_34_/L98H	ABPA	-	Systemic steroids	No follow up
12	60/M/2014	Sputum	VPCI	>16	>16	>8	>8	TR_34_/L98H	ABPA	-	Systemic steroids	No follow up

*^a^MIC, minimum inhibitory concentration; ITC, itraconazole; VRC, voriconazole; ISA, isavuconazole; POS, posaconazole; AMB, amphotericin B; ^b^Geometric mean MICs of all isolates against azole fungicides was 32 μg/ml except metconazole (3.8 μg/ml); ^c^FNAB, fine needle aspiration biopsy; ^d^VPCI, Vallabhbhai Patel Chest Hospital, Delhi; ^e^IPA, invasive pulmonary aspergillosis; ^f^COPD, chronic obstructive pulmonary disease; ^g^Hospital*

*1, Cancer Referral Hospital, Delhi; ^h^Hospital 2, General Medical Referral Hospital, Delhi; ^i^BAL, bronchoalveolar lavage; ^j^Hospital 3, Central Tuberculosis Hospital, Delhi; ^k^CPA, chronic pulmonary aspergillosis; ^l^ABPA, allergic bronchopulmonary aspergillosis.*

### Antifungal Susceptibility Testing (AFST)

All resistant *A. fumigatus* were subjected to AFST against four standard medical triazoles, amphotericin B (AMB), echinocandins, and 10 commonly used azole fungicides using CLSI M38-A2 broth microdilution Clinical and Laboratory Standards Institute [CLSI] (2008). The drugs tested included ITC (Lee Pharma, Hyderabad, India, and Janssen Research Foundation, Beerse, Belgium), VRC (Pfizer Central Research, Sandwich, Kent, UK), isavuconazole (ISA, Basilea Pharmaceutica International AG, Basel, Switzerland), posaconazole (POS, Merck, Whitehouse Station, NJ, USA), AMB (Sigma–Aldrich, Germany), caspofungin (CAS, Merck), micafungin (MFG, Astellas Toyama Co. Ltd., Japan), and anidulafungin (AFG, Pfizer). The tested azole fungicides were bromuconazole, cyproconazole, difenoconazole, epoxiconazole, penconazole, tebuconazole, triadimefon, metconazole, hexaconazole (Rallis India, Mumbai, India), and tricyclazole (Cheminova India, Mumbai, India). The AFST results were analyzed by using epidemiological cutoff values (ECVs) proposed by Espinel-Ingroff et al. (2011a,b, 2013) ITC, 1 μg/ml; VRC, 1 μg/ml; POS, 0.5 μg/ml; ISA, 1 μg/ml, AMB, 4 μg/ml, and CAS, 0.25 μg/ml (Pfaller et al., 2011).

### Mutation Analysis

To gain insight into the mechanisms responsible for azole resistance in *A. fumigatus*, isolates were subjected to amplification and sequencing of *cyp51A* gene along with the promoter region (Sharma et al., 2015). The amplified product was purified followed by sequencing on an ABI 3130XL genetic analyzer (Applied Biosystems, Foster City, CA, USA) using the BigDye terminator kit (v3.1, RR-100; Applied Biosystems; Chowdhary et al., 2013a). DNA sequences were analyzed with Sequencing Analysis software version 5.3.1 (Applied Biosystems). Consensus sequences were made using BioEdit software (version 7.0.5.3; Hall, 1999). The sequences of the resistant *A. fumigatus* strains were compared with the wild type susceptible reference *A. fumigatus* strain (Af293). The mutations in the resistant strains were further confirmed by mixed format real time PCR analysis as described previously (Klaassen et al., 2010).

### Microsatellite Typing

The genotypic relationship of the Indian resistant *A. fumigatus* isolates with the isolates from Asia, Middle East, and Europe harboring various mutations in *cyp51A* gene was determined by microsatellite typing using a panel of nine short tandem repeats (STR) as described previously (de Valk et al., 2005). The amplification of three di-, tri-, or tetranucleotide repeat markers was carried out using three multiplex PCRs, namely, M2, M3, and M4, respectively, (de Valk et al., 2005). Repeat numbers in each marker was assigned using *A. fumigatus* Af293 as reference. The STR data was imported to BioNumerics v5.1 software (Applied Maths, Sint-Martens-Latem, Belgium) for phylogenetic analysis. The dendrogram based on unweighted pair-group method with arithmetic mean (UPGMA) clustering using the Pearson correlation coefficient was generated. A total of 49 *A. fumigatus* isolates from Asia, Middle East, Europe, and Australia were included as controls to evaluate the genetic differences with the *A. fumigatus* isolates from India. The isolates from outside India were from following countries: China (clinical resistant, *n* = 8), Iran (environmental resistant, *n* = 5; environmental susceptible, *n* = 4), Kuwait (clinical resistant, *n* = 2; clinical susceptible, *n* = 2; environmental resistant, *n* = 8; environmental susceptible, *n* = 2), France (clinical resistant, *n* = 1), Germany (clinical resistant, *n* = 7; clinical susceptible, *n* = 1), the Netherlands (clinical resistant, *n* = 2; environmental resistant, *n* = 3), and Australia (clinical resistant, *n* = 2; clinical susceptible, *n* = 2). Also, Indian isolates (environmental resistant *n* = 3; environmental susceptible *n* = 6 and clinical susceptible *n* = 3) collected during the previous study on resistant *A. fumigatus* were included for comparison with the present clinical TRAF isolates from India (Chowdhary et al., 2012b).

## Results

Overall, during a 4-year survey period, 25.7% of clinical samples harbored *Aspergillus* species (*n* = 2117). Out of these 2117 isolates, 45.4% (*n* = 963) were *A. flavus*, followed by 32.4% (*n* = 685) *A. fumigatus*, 15.6% (*n* = 329) *Aspergillus* species and 6.6% (*n* = 140) *A. terreus* (Kathuria et al., 2015). Barring 12 isolates of *A. fumigatus* none of the other *Aspergillus* species grew on SDA plates supplemented with ITC and/or VRC. Of these, 11 *A. fumigatus* grew on both ITC and VRC supplemented SDA plates while a solitary isolate grew only on ITC plate. All the 12 resistant isolates were identified as *A. fumigatus sensu stricto* by β*-tubulin* and *calmodulin* genes sequencing.

Of the 12 resistant *A. fumigatus*, 11 showed a pan-azole resistant phenotype exhibiting high MIC of all the triazoles, namely, ITC [geometric mean (GM) MIC, 16 μg/ml], VRC (GM MIC, 8 μg/ml), ISA (GM MIC, 6.34 μg/ml), and POS (GM MIC, 2.82 μg/ml). In contrast, a solitary *A. fumigatus* isolate exhibited high MIC of only ITC (>16 μg/ml) and POS (1 μg/ml; **Table 1**). However, AMB (GM MIC, 0.4 μg/ml) and three echinocandins, namely, CAS, MFG, and AFG were active against all the resistant *A. fumigatus* isolates with GM MICs of 0.13 μg/ml, 0.017 μg/ml, and 0.02 μg/ml, respectively. Further all TRAF uniformly revealed cross-resistance to all the azole fungicides (MICs,>32 μg/ml) tested excepting metconazole (MICs, 3.80 μg/ml).

Overall, 1.75% (12/685) of the clinical *A. fumigatus* isolates were resistant. The major resistance mechanism observed among the eleven pan-azole resistant phenotype was TR_34_/L98H mutation (*n* = 10) and a solitary isolate exhibited three non-synonymous point mutations, namely, F46Y, D255E, and M172V. Another single point mutation, G54E, was observed in a solitary isolate that had high MICs to both ITC and POS.

**FIGURE 1:**
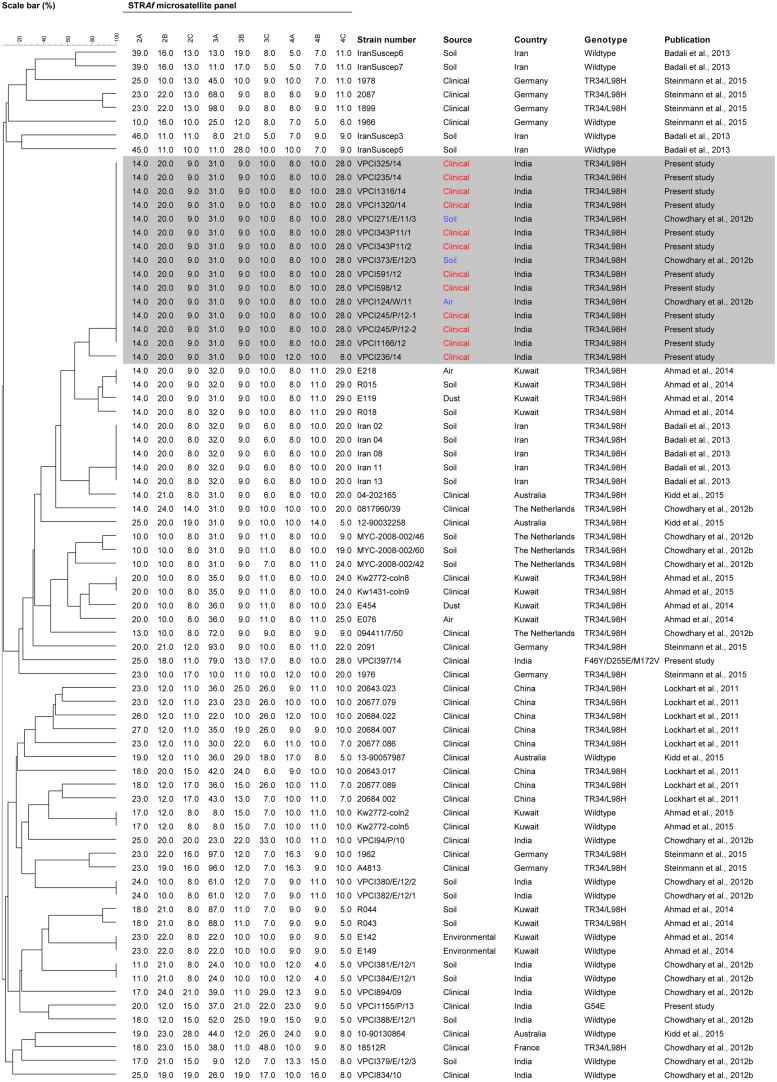
**Genotypicrelationship of Indian *Aspergillus fumigatus* isolates (clinical resistant, *n* = 14; clinical susceptible, *n* = 3; environmental resistant, *n* = 3; environmental susceptible, *n* = 6) with isolates from Asia including China (clinical resistant, *n* = 8), Middle East including Iran (environmental resistant, *n* = 5; environmental susceptible, *n* = 4), and Kuwait (clinical resistant, *n* = 2; clinical susceptible, *n* = 2; environmental resistant, *n* = 8; environmental susceptible, *n* = 2), and Europe including France (clinical resistant, *n* = 1), Germany (clinical resistant, *n* = 7; clinical susceptible, *n* = 1), the Netherlands (clinical resistant, *n* = 2; environmental susceptible, *n* = 3), and Australia (clinical resistant, *n* = 2; clinical susceptible, *n* = 2)**. The dendrogram is based on a categorical analysis of nine microsatellite markers in combination with Unweighted Pair Group Method with arithmetic mean clustering. The scale bar indicates the percentage identity. The isolates VPCI 343/P/11/1, VPCI 343/P/11/2, and VPCI 245/P/12-1, VPCI 245/P/12-2 were serial isolates from two individual patients, respectively.

The STR typing data revealed a single microsatellite complex (MC) among all the TR_34_/L98H genotypes whereas solitary isolate each of G54E and non-synonymous mutant of *A. fumigatus* represented two distinct genotypes (**Figure 1**). The TR_34_/L98H MC was homogenous and shared all the nine loci except one isolate that differed at two loci (4A and 4C). Further to determine the genetic relatedness among the present Indian TR_34_/L98H clinical isolates (*n* = 10) comparison with Indian TR_34_/L98H environmental isolates (*n* = 3) collected from the previous study was done (Chowdhary et al., 2012b). Both the environmental and clinical *A. fumigatus* isolates had an identical STR pattern. The genotypes of all Indian TR_34_/L98H isolates were distinct from the TR_34_/L98H strains of Chinese, Kuwait, Iran, and European isolates (Lockhart et al., 2011; Badali et al., 2013; Ahmad et al., 2014, 2015; Steinmann et al., 2015). However, environmental *A. fumigatus* isolates from Kuwait (*n* = 4) and Iran (*n* = 5) were more closely related with Indian TRAF isolates with similarity observed at 5–6 of the nine loci studied. Although the Chinese clinical TRAF isolates (*n* = 8) also formed a separate cluster but unlike Indian isolates they were markedly heterogeneous exhibiting variable STR patterns. The STR typing data of the clinical Kuwait TRAF isolates (*n* = 6) revealed homogeneity among them, however, when compared to Indian TRAF clinical isolates they showed differences at 6–7 loci (Ahmad et al., 2015).

The detailed clinical record of all patients whose clinical samples yielded TRAF was retrieved from the database. The majority of patients were diagnosed as invasive aspergillosis (IA, *n* = 5) followed by allergic bronchopulmonary aspergillosis (ABPA, *n* = 4) and chronic pulmonary aspergillosis (CPA, *n* = 3). The most common underlying condition among the IA patients was chronic obstructive pulmonary disease (COPD) in three patients and myelodysplastic syndrome and pulmonary adenocarcinoma in the remaining two patients, respectively. IA was defined as probable or proven according to the criteria of the European Organization for Research and Treatment of Cancer/Mycoses Study Group (Ascioglu et al., 2002; De Pauw et al., 2008). All cases of IA in the present study were proven cases and FNAB/lung biopsy or resected sinus tissue was positive for fungus and yielded *A. fumigatus* in culture. IPA (*n* = 4) was the most common manifestation and a solitary case had invasive rhino-cerebral sinusitis. All COPD patients who finally developed IA during the course of 5–10 years of illness had been admitted to the medical units or ICU repeatedly (4–9 episodes per year) for respiratory symptoms with severe airflow obstruction. All these patients were on systemic and inhaled steroids for a long period ranging from 10 to 12 years. Associated co-morbidities like diabetes mellitus and tuberculosis were observed in two of the three COPD patients. Allergic aspergillosis manifesting as ABPA was diagnosed in four patients by a combination of clinical, mycoserologic, and radiological features (Agarwal et al., 2013). None of the ABPA patients diagnosed in the present study were on azoles when their culture grew TRAF. The third clinical entity was CPA, which was diagnosed by chronic duration of clinical symptoms (>3 months), progressive pulmonary lesions with or without cavitation, precipitating antibodies to *A. fumigatus* in serum, and mycological evidence of fungal presence. Further, none of the patients harboring identical TR_34_/L98H genotype (*n* = 10) admitted to the Chest hospital (*n* = 8) in the present study had an overlapping time frame during their admission. Also, two patients harboring the same genotype were from two different hospitals in Delhi (**Table 1**).

Overall, the mortality was highest in IA (four of five patients) and CPA (two of three patients) and a solitary case of ABPA also had a fatal outcome. Among IA patients, VRC (6 mg/kg intravenous 12 hourly followed by 4 mg/kg and switched to oral 200 mg twice a day when clinically stable) was administered in three patients and two were started on AmB deoxycholate (1 mg/kg/day). All CPA patients were given VRC for 7–10 days before the antifungal susceptibility results were conveyed. The two CPA patients had severe lung involvement and the disease was fatal in both of them within 1 week. However, in one case therapy was switched to amphotericin B for one month followed by repeat BAL culture that yielded negative results for *Aspergillus* and the patient was discharged on oral VRC on follow up. However, patient failed to report for the follow up visits. The patients with ABPA in the present study were managed symptomatically and systemic steroids were the mainstay of therapy.

## Discussion

In the present study, we examined azole susceptibility and resistant mechanisms among *A. fumigatus* isolates from patients with bronchopulmonary aspergillosis in a referral Chest Institute, which caters to a vast population of Delhi and adjoining states of Uttar Pradesh, Haryana and also to far remote regions of India. The rate of azole resistance in *A. fumigatus* isolates in our study was 1.75% (12/685) during a 4-year period, which is remarkably low compared to the high prevalence in Europe including UK (6.6–27.8%), the Netherlands (3.1–4.6%), and Germany (3.2%; Snelders et al., 2008; Howard et al., 2009; Bueid et al., 2010; van der Linden et al., 2011; Bader et al., 2013). In contrast in Spain, a lower prevalence rate (2.5%) of azole resistance in *A. fumigatus* complex has been reported suggesting that azole resistance has not yet uniformly spread in Europe (Escribano et al., 2013). TR_34_/L98H was the predominant resistance mechanism in 83.3% of the Indian TRAF isolates followed by G54E (*n* = 1) and a non-synonymous mutation (*n* = 1).

Short tandem repeats typing of the TRAF isolates demonstrated a single cluster, with a homogenous population. It is also worth mentioning here that the patients’ positive for TRAF isolates in the present study were both from Delhi, and other states namely West Bengal, Haryana, and Uttar Pradesh. These states previously have been found to harbor *A. fumigatus* isolates carrying TR_34_/L98H in soil samples (Chowdhary et al., 2012b). Notably the genotype detected in clinical isolates in this study was identical to the TR_34_/L98H genotype reported earlier from environmental samples from India and shared the identical nine loci, suggesting environmental origin of this major resistance mechanism (Chowdhary et al., 2012b). This is the first study outside the Netherlands revealing the possibility of acquisition of clinical isolates linked with the environment. However, in contrast to the heterogeneity observed in environmental and clinical isolates in the Netherlands (Klaassen et al., 2012), the Indian TRAF in the present study had a homogenous population which was recently also confirmed with whole genome sequencing (Abdolrasoulia et al., 2015).

**Table 2** summarizes the reports of TRAF isolation harboring mutations in the *cyp51A* gene from clinical and environmental sources in Asia and the neighboring Middle East. The initial reports describing azole resistance in clinical *A. fumigatus* isolates originated from Europe in the late 1990s followed by systematical investigations in several European countries which, reported prevalence, mechanism, and genomic aspects of TRAF (Mellado et al., 2007; Verweij et al., 2007; Rodriguez-Tudela et al., 2008; Howard et al., 2009; Mortensen et al., 2010; Burgel et al., 2012; Morio et al., 2012; Bader et al., 2013; Astvad et al., 2014; Fischer et al., 2014; Spiess et al., 2014; van der Linden et al., 2015). However, the first comprehensive report on the occurrence of TRAF isolates in Asia originated from China during 2008–2009 from the ARTEMIS global sentinel surveillance program demonstrating TR_34_/L98H resistance mechanism in 27.5% (8/29) *A. fumigatus* isolates (Lockhart et al., 2011; **Table 2**). In contrast, more recently few studies from Japan, described TRAF in clinical isolates but interestingly none of them exhibited TR_34_/L98H resistance mechanism, instead several SNPs and novel mutations, F332K and P216L were reported (Asano et al., 2011; Hagiwara et al., 2014). The single center study from Japan reported 5.2% (1/19) TRAF isolates harboring only G54E/R/W and I266N mutation (Tashiro et al., 2012a,b). Notably, a recent study on environmental sampling of air from a pumpkin farm sprayed with azole fungicides, at Nihon University, Japan was carried out by using an outdated and less efficacious settle plate method and reported no azole resistance in 50 *A. fumigatus* isolates (Kano et al., 2015). The lack of isolation of TRAF could be attributed to not adopting a highly efficacious sampling method such as using an air sampler and to the low number of isolates tested for resistance resulting in false rates of TRAF prevalence in the environment. The possibility of missing resistance was recently also highlighted in the SCARE study (van der Linden et al., 2015). Further, planned studies with wider coverage areas and different sources such as soil samples, air, wooden debris etc., should be undertaken for finding out the true prevalence of TRAF isolates. In India, a comprehensive wider environmental survey covering north, south, and eastern India analyzed 486 environmental samples comprising soil from flowerbeds of nurseries, surrounding parks of hospitals, cotton trees, tea gardens, paddy fields, soil containing bird excreta, and decayed wood of tree trunks revealed 7% TRAF isolates in the environment carrying TR_34_/L98H mutation (Chowdhary et al., 2012b). Also, a recently described VRC resistant TR_46_/Y121F/T289A mechanism was observed in environmental *A. fumigatus* isolates from agricultural fields in India (Chowdhary et al., 2014a). Barring the solitary environmental report on TR_46_/Y121F/T289A mechanism from India none of the clinical or environmental samples from Asia has yet documented the occurrence of *A. fumigatus* isolates carrying this mechanism so far. However, considering the presence of this new resistant mechanism in the environment of India it may be anticipated that in future it may spread to the neighboring Asian countries. In addition to the above-discussed reports on triazole resistance in Asia, the neighboring Middle East countries, Iran, and Kuwait had reported 12.2 and 7% TRAF carrying TR_34_/L98H resistance in the environment, respectively, (Badali et al., 2013; Ahmad et al., 2014). A similar range of resistance prevalence of 3.2 and 12.5% TR_34_/L98H *A. fumigatus* isolates was observed in clinical samples from CPA and ABPA patients from Iran and Kuwait, respectively, (Seyedmousavi et al., 2013; Ahmad et al., 2015). Based on the fact that the Indian TRAF isolates exhibited a distinct cluster away from the Chinese TR_34_/L98H isolates, it may be suggested that resistance among the *A. fumigatus* strains across Asia has evolved from separate strains and not from a common resistant ancestor, which may have spread worldwide.

**Table 2:** Distribution of TRAF in clinical and environmental samples in Asia and Middle East harboring mutations in the *Cyp51A* gene.

Country	Resistance mechanism	Source	Resistance rate (No. of resistant *A. fumigatus* / No. of *A. fumigatus* tested)	Reference
China	M220I, G54R	Clinical	4/6 (66.6%)^a^	Chen et al. (2005); Xu et al. (2010)
	TR34/L98H	Clinical	8/29 (27.5%)	Lockhart et al. (2011)
	SNP^b^	Environmental, Poultry	11^c^/175	Wang et al. (2014)
India	TR34/L98H	Clinical	2/103 (1.9%)	Chowdhary et al. (2012a)
	TR34/L98H	Environmental	44/630 (7%)	Chowdhary et al. (2012b)
	TR34/L98H & TR46/Y121F/T289A	Environmental	8/126 (6.3%) 6/126 (4.8%)	Chowdhary et al. (2014a)
	TR34/L98H & G54E	Environmental	4/5 (80%) 1/5 (20%)	Sharma et al. (2015)
Iran	TR34/L98H	Clinical	4/124 (3.2%)	Seyedmousavi et al. (2013)
	TR34/L98H	Environmental	5/41 (12.1%)	Badali et al. (2013)
Japan	F332K	Clinical	1/19 (5.2%)	Asano et al. (2011)
	G54E/R/W and I266N	Clinical	12/196 (6.1%)	Tashiro et al. (2012a; 2012b)
	P216L	Clinical	1/8 (12.5%)	Hagiwara et al. (2014)
Kuwait	TR34/L98H	Environmental	8/115 (7%)	Ahmad et al. (2014)
	TR34/L98H	Environmental	1/50 (2%)	Ahmad et al. (2015)
	TR34/L98H	Clinical	2/16 (12.5%)	Ahmad et al. (2015)
Taiwan	Not mentioned^d^	Clinical	2/40 (5%)	Hsueh et al. (2005)

*^a^serial isolates from a patient suffering from lung aspergilloma; ^b^SNP, single nucleotide polymorphisms such asF46Y, M172V, N248T, N248K, D255E, and E427K also present in susceptible *A. fumigatus* strains were detected; ^c^total no. of *A. fumigatus* obtained from French and Chinese avian farms; ^d^The mutation in *cyp51A* gene was not investigated.*

It is also pertinent to mention that the high rates of triazole resistance in Europe as compared to Asia could be due to environmental factors or more so by frequent use of azoles in clinical settings and in the environment. Azole fungicides are extensively used in agriculture for crop protection to control mildews and rusts of grains, fruits, vegetables, and also for preservation of materials like wood etc. Geographically, Europe is the dominant market where fungicide usage is significantly high in production of fruits and vegetables along with wheat and vineyard (Stensvold et al., 2012). Thus, the reports of high resistance rates in environmental *A. fumigatus* isolates from the Netherlands, Belgium, UK, and Germany can be attributed to higher fungicide usage. Similarly, the absence or very low prevalence of triazole resistance in *A. fumigatus* in the USA could possibly be due to the low usage of fungicides in the USA as compared to Europe ^1^. Notably, triazole resistance was screened among 1026 *A. fumigatus* isolates from 22 states of USA and none had TR_34_/L98H mutation (Pham et al., 2014). In India, the crop protection chemicals account for ∼2% of the total chemicals market and currently India is the second largest manufacturer of pesticides in Asia, second only to Japan^2^. Among different classes of pesticides used in India the percent share of insecticides (60%) is highest followed by fungicides (19%), herbicides (16%), biopesticides (3%), and others (3%). Per capita consumption of crop protection products in India is 0.6 kg/ha compared to 16 kg/ha in Taiwan, 13 kg/ha in China, and 12 kg/ha in Japan^2^. Finally, the environmental resistance mechanism in *A. fumigatus* isolates have been reported from two highly populated countries in Asia, i.e., China and India. Further, the environment of agricultural fields in India has been harboring variable *cyp51A* mediated resistant patterns in *A. fumigatus* isolates, which are continuously exposed to agricultural azole fungicides. It is emphasized that in depth analysis of azole resistance in *A. fumigatus* isolates in both clinical laboratories and environmental settings is required to prevent its spread and emergence in neighboring Asian countries.

## Conflict of Interest Statement

Jacques F. Meis received grants from Astellas, Basilea, and Merck. He has been a consultant to Astellas, Basilea, and Merck and received speaker’s fees fromMerck and Gilead. All other authors: no potential conflicts of interest. The authors alone are responsible for the content and writing of the paper.

## Footnotes

1 http://ec.europa.eu/food/fs/sc/ssc/out278_en.pdf

2 http://www.ficci.com/spdocument/20292/petro1.pdf
